# Supplementary material for: Understanding Colour Tuning Rules and Predicting Absorption Wavelengths of Microbial Rhodopsins by Data-Driven Machine-Learning Approach
Source: Sci Rep. 2018 Oct 22;8:15580. doi: 10.1038/s41598-018-33984-w (PMC6197263; doi:10.1038/s41598-018-33984-w)
Supplement: Supplementary file 1 — Supplementary information main text [file 41598_2018_33984_MOESM1_ESM.pdf]

Supplementary Information of "Karasuyama et al. Understanding Colour Tuning Rules and Predicting Absorption Wavelengths of Microbial Rhodopsins by Data-Driven Machine-Learning Approach" submitted to Scientific Reports

Masayuki Karasuyama[1,2,3], Keiichi Inoue[4,2,5,6], Ryoko Nakamura[7], Hideki Kandori[4,5], and Ichiro Takeuchi[1,3,7]

[1]Department of Computer Science, Nagoya Institute of Technology, Gokiso, Showa-ku, Nagoya, Aichi, 466-8555, Japan
[2]PRESTO, Japan Science and Technological Agency (JST), 4-1-8 Honcho, Kawaguchi, Saitama, 332-0012, Japan
[3]Center for Materials research by Information Integration, National Institute for Materials Science (NIMS), Tsukuba 305-0047, Japan
[4]Department of Life Science and Applied Chemistry, Nagoya Institute of Technology, Gokiso, Showa-ku, Nagoya, Aichi, 466-8555, Japan
[5]OptoBioTechnology Research Center, Gokiso, Showa-ku, Nagoya, Aichi, 466-8555, Japan
[6]The Institute for Solid State Physics, The University of Tokyo, 5-1-5 Kashiwanoha, Kashiwa, Chiba 277-8581, Japan.
[7]RIKEN Center for Advanced Intelligence Project, Nihonbashi 1-chome, 1-4-1 Nihonbashi, Chuo-ku, Tokyo 103-0027, Japan

Supplementary Information 1: Abbreviations of the names of microbial rhodopsins

*Acet*R1: *acetabularia* rhodopsin of *Acetabularia acetabulum*

AR1: archaerhodopsin 1 of *Halorubrum sodomense*

AR2: archaerhodopsin 2 of *Halorubrum sodomense*

AR3: archaerhodopsin 3 of *Halorubrum sodomense*

ASR: *Anabaena* sensory rhodopsin of *Anabaena (Nostoc) sp.* PCC7120

BPR: blue proteorhodopsin of uncultured γ-proteobacterium

BR: bacteriorhodopsin of *Halobacterium salinarum*

Chrimson: channelrhodopsin-1 of *Chlamydomonas noctigama*

Chronos: channelrhodopsin of *Stigeoclonium helveticum*

*Cn*ChR2: channelrhodopsin-2 of *Chlamydomonas noctigama*

*Cr*ChR1: channelrhodopsin-1 of *Chlamydomonas reinhardtii*

*Cr*ChR2: channelrhodopsin-2 of *Chlamydomonas reinhardtii*

*D*ChR1: channelrhodopsin-1 of *Dunaliella salina*

*Pa*R: DTG rhodopsin of *Pantoea ananatis*

*Psp*R: DTG rhodopsin of *Pseudomonas putida*

*Pv*R: DTG rhodopsin of *Pantoea vagans*

ESR: proton pump rhodopsin of *Exiguobacterium sibiricum*

FR: chloride pump rhodopsin of *Fulvimarina pelagi*

*Gl*NaR: sodium pump rhodopsin of *Gillisia limnaea*

GPR: green proteorhodopsin of uncultured γ-proteobacterium

GR: *Gloeobacter* rhodopsin of *Gloeobacter violaceus* PCC 7421

*Gt*ACR1: anion channel rhodopsin of *Guillardia theta*

*Gt*ACR2: anion channel rhodopsin of *Guillardia theta*

*Hm*BRI: bacteriorhodopsin of *Haloarcula marismortui*

*Hm*BRII: bacteriorhodopsin of *Haloarcula marismortui*

*Hm*HR: halorhodopsin of *Haloarcula marismortui*

*Hm*SRI: sensory rhodopsin I of *Haloarcula marismortui*

*Hm*SRII: sensory rhodopsin II of *Haloarcula marismortui*

*Hm*SRIII: sensory rhodopsin III of *Haloarcula marismortui*

*Hs*HR: halorhodopsin of *Halobacterium salinarum*

*Hs*SRI: sensory rhodopsin I of *Halobacterium salinarum*

*Hv*SRI: sensory rhodopsin I of *Haloarcula vallismortis*

*Hw*BR: bacteriorhodopsin of *Haloquadratum walsbyi*

*Ia*NaR: sodium pump rhodopsin of *Indibacter alkaliphilus*

*Ia*R1: proteorhodopsin of *Indibacter alkaliphilus*

KR1: proteorhodopsin of *Krokinobacter eikastus*

KR2: sodium pump rhodopsin of *Krokinobacter eikastus*

*La*NaR: sodium pump rhodopsin of *Lyngbya aestuarii*

LR: *Leptosphaeria* rhodopsin of *Leptosphaeria maculans*

MR: middle rhodopsin of *Haloquadratum walsbyi*

*Nd*PR: proteorhodopsin of *Nonlabens dokdonensis*

*Nd*NaR: sodium pump rhodopsin of *Nonlabens dokdonensis*

*Nm*ClR: chloride pump rhodopsin of *Nonlabens marinus*

*Nm*NaR: sodium pump rhodopsin of *Nonlabens marinus*

*Nm*PR: proteorhodopsin of *Nonlabens marinus*

*Np*HR: halorhodopsin of *Natronobacterium pharaonis*

*Np*SRII: sensory rhodopsin II of *Natronobacterium pharaonis*

NR: *Neurospora* rhodopsin of *Neurospora crassa*

*Phaeo*RD1: putative proton pump rhodopsin of *Phaeosphaeria* (*Stagonospora*) *nodorum*

*Phaeo*RD2: putative proton pump rhodopsin of *Phaeosphaeria* (*Stagonospora*) *nodorum*

*Po*XeR: xenorhodopsin of *Parvularcula oceani*

*Ps*ChR2: channelrhodopsin-2 of *Proteomonas sulcata*

*Sr*SRI: sensory rhodopsin I of *Salinibacter ruber*

TR: thermophilic rhodopsin of *Thermus thermophilus*

*Ts*ChR: channelrhodopsin of *Tetraselmis striata*

XR: xanthorhodopsin of *Salinibacter ruber*

Supplementary Information 2: References for Supplementary Table 1

See MS Word file `References_Supplementary_Table_1.docx`

Supplementary Information 3: Software implementation

Our R code for the group-LASSO-based wavelength prediction is available at `http://...` (The site will be public after acceptance). "R" is a standard command-based environment for statistical computations that is freely available and installable on Windows, Mac OS, and most major distributions of Linux. In the R command-line, our package can be installed by the following command at the directory in which the downloaded file exists:

```
> install.packages(c("grplasso", "seqinr", "foreach"))
> install.packages("GrplassoSeq_1.0.tar.gz",
                   repos = NULL, type = "source")
```

A set of sequences and their wavelengths should be provided as two separated input files. Sequence data should be in the FASTA format, which can be read by the `read.fasta` function of the R `seqinr` package (`https://cran.r-project.org/web/packages/seqinr/index.html`). Note that, for both the training and target proteins, the sequences should be identically arranged according to length beforehand. Wavelengths should be stored in a text file in which each line contains a wavelength for a sequence. The order of sequences in the FASTA file and the wavelength file should be consistent. For wavelength-unknown sequences, please specify "NA" in the wavelength file. Proteins specified as "NA" are regarded as target proteins, and other proteins with wavelengths are used as training proteins.

Supplementary Table 1: Microbial rhodopsin database composed of the amino-acid sequences and absorption wavelengths λ_{max}s of 519 proteins previously reported in the literature and 277 proteins newly investigated by our group.

See MS Excel file: `Supplementary_Table_1.xlsx`

Supplementary Table 2: List of all fitted coefficient parameters

See MS Excel file: `Supplementary_Table_2.xlsx`

Supplementary Figure 1

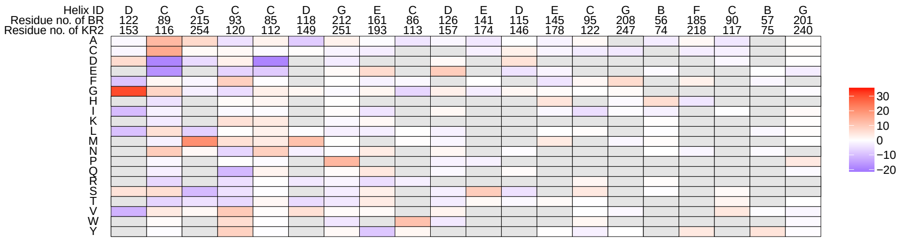

Figure S1: **Coefficient parameters of the statistical model fitted with all 796 rhodopsin proteins as the training set**. The coefficient parameters at 20 active residues in decreasing order of s_j are given. This figure has the same format as Figure 5, except that all 796 rhodopsin proteins, including those in the KR2 group, were used as the training data. Although the resulting coefficient parameters depend on the given training data, most of the coefficients parameters in Supplementary Figure S3 are similar to those in Figure 5 in the main text. The correlation coefficient between these two results was 0.9650.

Supplementary Figure 2

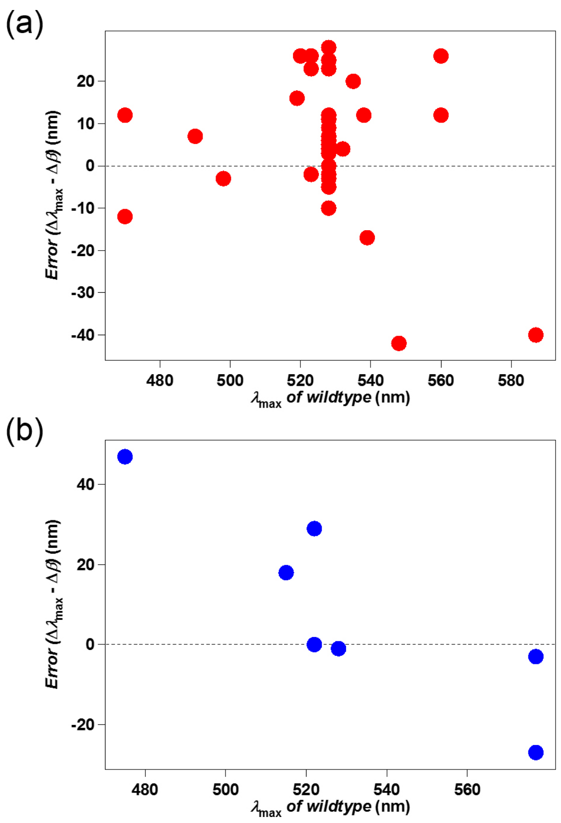

Figure S2: **The correlation of the difference between the experimentally observed absorption shift ($\lambda_{\max}$) and predicted shift by ML ($\Delta\beta$) against the $\lambda_{\max}$ of wildtype protein for the removal (a) or introduction (b) of counterion (D or E) at homologous positions to BR 85 or 89.**

Supplementary Figure 3

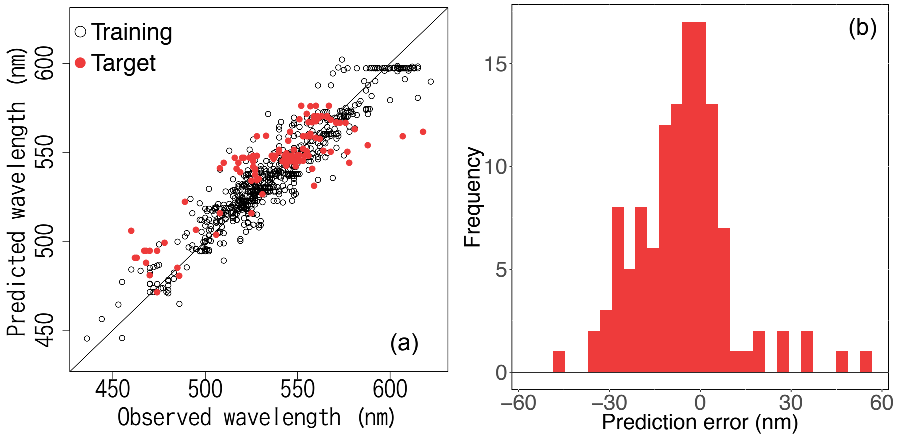

Figure S3: **Absorption wavelength prediction results for GR wildtype and its 121 variants.** The plots (a) and (b) have the same format as Figure 6 (e) and (f). GR wildtype and its 121 variants are the target proteins and the other 674 proteins are contained in the training data. The mean absolute error for the target proteins was 12.4 nm, and the correlation coefficient ρ was $\rho = 0.8596$ (p-value for the null hypothesis $\rho = 0$ was 8.7154×10^{-37}).
