# Supplementary material for: Understanding Colour Tuning Rules and Predicting Absorption Wavelengths of Microbial Rhodopsins by Data-Driven Machine-Learning Approach
Source: Sci Rep. 2018 Oct 22;8:15580. doi: 10.1038/s41598-018-33984-w (PMC6197263; doi:10.1038/s41598-018-33984-w)
Supplement: Supplementary file 2 — References for Supplementary Table 1 [file 41598_2018_33984_MOESM2_ESM.docx]

**Supplementary Information for**

“Understanding Colour Tuning Rules and Predicting Absorption Wavelengths of Microbial Rhodopsins by Data-Driven Machine-Learning Approach”

Masayuki Karasuyama^1,2,3^, Keiichi Inoue^4,2,5,6^, Ryoko Nakamura^7^,

Hideki Kandori^4,5^, and Ichiro Takeuchi^1,3,7^

^1^Department of Computer Science, Nagoya Institute of

Technology, Gokiso, Showa-ku, Nagoya, Aichi, 466-8555, Japan

^2^PRESTO, Japan Science and Technological Agency (JST), 4-1-8

Honcho, Kawaguchi, Saitama, 332-0012, Japan

^3^Center for Materials research by Information Integration, National

Institute for Materials Science (NIMS), Tsukuba 305-0047, Japan

^4^Department of Life Science and Applied Chemistry, Nagoya

Institute of Technology, Gokiso, Showa-ku, Nagoya, Aichi,

466-8555, Japan

^5^OptoBioTechnology Research Center, Gokiso, Showa-ku, Nagoya,

Aichi, 466-8555, Japan

^6^The Institute for Solid State Physics, The University of Tokyo,

5-1-5 Kashiwanoha, Kashiwa, Chiba 277-8581, Japan.

^7^RIKEN Center for Advanced Intelligence Project, Nihonbashi

1-chome, 1-4-1 Nihonbashi, Chuo-ku, Tokyo 103-0027, Japan

**References in Supplementary Table 1**

1 Oesterhelt, D. & Stoeckenius, W. Isolation of the cell membrane of *Halobacterium halobium* and its fractionation into red and purple membrane. *Methods Enzymol* **31**, 667-678, (1974).

2 Sudo, Y. *et al.* A blue-shifted light-driven proton pump for neural silencing. *J. Biol. Chem.* **288**, 20624-20632, (2013).

3 Kawanabe, A., Furutani, Y., Jung, K. H. & Kandori, H. Engineering an inward proton transport from a bacterial sensor rhodopsin. *J. Am. Chem. Soc.* **131**, 16439-16444, (2009).

4 Inoue, K. *et al.* A light-driven sodium ion pump in marine bacteria. *Nat. Commun.* **4**, 1678, (2013).

5 Yoshizawa, S. *et al.* Functional characterization of flavobacteria rhodopsins reveals a unique class of light-driven chloride pump in bacteria. *Proc. Natl. Acad. Sci. USA* **111**, 6732-6737, (2014).

6 Balashov, S. P. *et al.* Light-driven Na^+^ pump from *Gillisia limnaea*: a high-affinity Na^+^ binding site is formed transiently in the photocycle. *Biochemistry* **53**, 7549-7561, (2014).

7 Bertsova, Y. V., Bogachev, A. V. & Skulachev, V. P. Proteorhodopsin from *Dokdonia* sp. PRO95 is a light-driven Na^+^-pump. *Biochemistry (Mosc)* **80**, 449-454, (2015).

8 Kato, H. E. *et al.* Structural basis for Na^+^ transport mechanism by a light-driven Na^+^ pump. *Nature* **521**, 48-53, (2015).

9 Inoue, K., Konno, M., Abe-Yoshizumi, R. & Kandori, H. The Role of the NDQ Motif in Sodium-Pumping Rhodopsins. *Angew. Chem. Int. Ed.* **54**, 11536-11539, (2015).

10 Inoue, K., Nomura, Y. & Kandori, H. Asymmetric functional conversion of eubacterial light-driven ion pumps. *J. Biol. Chem.* **291**, 9883-9893, (2016).

11 Inoue, K. *et al.* A natural light-driven inward proton pump. *Nat. Commun.* **7**, 13415, (2016).

12 Hegemann, P. O., D. & Bamberg, E. The transport activity of the light-driven chloride pump halorhodopsin is regulated by green and blue light. *Biochim Biophys Acta* **819**, 195-205, (1985).

13 Rüdiger, M., Haupts, U., Gerwert, K. & Oesterhelt, D. Chemical reconstitution of a chloride pump inactivated by a single point mutation. *EMBO J.* **14**, 1599-1606, (1995).

14 Rüdiger, M. & Oesterhelt, D. Specific arginine and threonine residues control anion binding and transport in the light-driven chloride pump halorhodopsin. *EMBO J* **16**, 3813-3821, (1997).

15 Sato, M. *et al.* Ser-130 of *Natronobacterium pharaonis* halorhodopsin is important for the chloride binding. *Biophys. Chem.* **104**, 209-216, (2003).

16 Sato, M. *et al.* Role of putative anion-binding sites in cytoplasmic and extracellular channels of *Natronomonas pharaonis* halorhodopsin. *Biochemistry* **44**, 4775-4784, (2005).

17 Hasegawa, C. *et al.* Interaction of the halobacterial transducer to a halorhodopsin mutant engineered so as to bind the transducer: Cl^-^ circulation within the extracellular channel. *Photochem. Photobiol.* **83**, 293-302, (2007).

18 Sudo, Y. *et al.* A microbial rhodopsin with a unique retinal composition shows both sensory rhodopsin II and bacteriorhodopsin-like properties. *J Biol Chem* **286**, 5967-5976, (2011).

19 Ihara, K., Amemiya, T., Miyashita, Y. & Mukohata, Y. Met-145 is a key residue in the dark adaptation of bacteriorhodopsin homologs. *Biophys. J.* **67**, 1187-1191, (1994).

20 Feng, J. *et al.* Genetic cloning and functional expression in *Escherichia coli* of an archaerhodopsin gene from *Halorubrum xinjiangense*. *Extremophiles* **10**, 29-33, (2006).

21 Saint Clair, E. C. *et al.* Near-IR resonance Raman spectroscopy of archaerhodopsin 3: effects of transmembrane potential. *J. Phys. Chem. B* **116**, 14592-14601, (2012).

22 Inoue, K. *et al.* Converting a light-driven proton pump into a light-gated proton channel. *J. Am. Chem. Soc.* **137**, 3291-3299, (2015).

23 Ikeda, D., Furutani, Y. & Kandori, H. FTIR study of the retinal Schiff base and internal water molecules of proteorhodopsin. *Biochemistry* **46**, 5365-5373, (2007).

24 Dioumaev, A. K. *et al.* Proton transfers in the photochemical reaction cycle of proteorhodopsin. *Biochemistry* **41**, 5348-5358, (2002).

25 Man, D. *et al.* Diversification and spectral tuning in marine proteorhodopsins. *EMBO J.* **22**, 1725-1731, (2003).

26 Wang, W. W., Sineshchekov, O. A., Spudich, E. N. & Spudich, J. L. Spectroscopic and photochemical characterization of a deep ocean proteorhodopsin. *J. Biol. Chem.* **278**, 33985-33991, (2003).

27 Man-Aharonovich, D. *et al.* Characterization of RS29, a blue-green proteorhodopsin variant from the Red Sea. *Photochem. Photobiol. Sci.* **3**, 459-462, (2004).

28 Sabehi, G. *et al.* New insights into metabolic properties of marine bacteria encoding proteorhodopsins. *PLoS Biol.* **3**, e273, (2005).

29 Rangarajan, R., Galan, J. F., Whited, G. & Birge, R. R. Mechanism of spectral tuning in green-absorbing proteorhodopsin. *Biochemistry* **46**, 12679-12686, (2007).

30 Kralj, J. M. *et al.* Protonation state of Glu142 differs in the green- and blue-absorbing variants of proteorhodopsin. *Biochemistry* **47**, 3447-3453, (2008).

31 Yoshitsugu, M., Shibata, M., Ikeda, D., Furutani, Y. & Kandori, H. Color change of proteorhodopsin by a single amino acid replacement at a distant cytoplasmic loop. *Angew Chem Int Ed Engl* **47**, 3923-3926, (2008).

32 Kralj, J. M., Spudich, E. N., Spudich, J. L. & Rothschild, K. J. Raman spectroscopy reveals direct chromophore interactions in the Leu/Gln105 spectral tuning switch of proteorhodopsins. *J. Phys. Chem. B* **112**, 11770-11776, (2008).

33 Bergo, V. B. *et al.* His-75 in proteorhodopsin, a novel component in light-driven proton translocation by primary pumps. *J. Biol. Chem.* **284**, 2836-2843, (2009).

34 Yoshitsugu, M., Yamada, J. & Kandori, H. Color-changing mutation in the E-F loop of proteorhodopsin. *Biochemistry* **48**, 4324-4330, (2009).

35 Yamada, K., Kawanabe, A. & Kandori, H. Importance of alanine at position 178 in proteorhodopsin for absorption of prevalent ambient light in the marine environment. *Biochemistry* **49**, 2416-2423, (2010).

36 Maiti, T. K., Yamada, K., Inoue, K. & Kandori, H. L105K mutant of proteorhodopsin. *Biochemistry* **51**, 3198-3204, (2012).

37 Janke, C. *et al.* Photocycle and vectorial proton transfer in a rhodopsin from the eukaryote *Oxyrrhis marina*. *Biochemistry* **52**, 2750-2763, (2013).

38 Bogomolni, R. A. & Spudich, J. L. The photochemical reactions of bacterial sensory rhodopsin-I. Flash photolysis study in the one microsecond to eight second time window. *Biophysical J.* **52**, 1071-1075, (1987).

39 Jung, K. H., Spudich, E. N., Dag, P. & Spudich, J. L. Transducer-binding and transducer-mutations modulate photoactive-site-deprotonation in sensory rhodopsin I. *Biochemistry* **38**, 13270-13274, (1999).

40 Kitajima-Ihara, T. *et al.* Salinibacter sensory rhodopsin: sensory rhodopsin I-like protein from a eubacterium. *J Biol Chem* **283**, 23533-23541, (2008).

41 Suzuki, D. *et al.* Effects of chloride ion binding on the photochemical properties of salinibacter sensory rhodopsin I. *J. Mol. Biol.* **392**, 48-62, (2009).

42 Yagasaki, J. *et al.* Spectroscopic studies of a sensory rhodopsin I homologue from the archaeon Haloarcula vallismortis. *Biochemistry* **49**, 1183-1190, (2010).

43 Sudo, Y., Yuasa, Y., Shibata, J., Suzuki, D. & Homma, M. Spectral tuning in sensory rhodopsin I from *Salinibacter ruber*. *J. Biol. Chem.* **286**, 11328-11336, (2011).

44 Irieda, H. *et al.* Structural characteristics around the beta-ionone ring of the retinal chromophore in Salinibacter sensory rhodopsin I. *Biochemistry* **50**, 4912-4922, (2011).

45 Tsukamoto, T., Demura, M. & Sudo, Y. Irreversible trimer to monomer transition of thermophilic rhodopsin upon thermal stimulation. *J Phys Chem B* **118**, 12383-12394, (2014).

46 Tsukamoto, T. *et al.* X-ray Crystallographic Structure of Thermophilic Rhodopsin: implications for high thermal stability and optogenetic function. *J. Biol. Chem.* **291**, 12223-12232, (2016).

47 Lee, K. A. *et al.* Mistic-fused expression of algal rhodopsins in *Escherichia coli* and its photochemical properties. *Biochim. Biophys. Acta* **1850**, 1694-1703, (2015).

48 Muroda, K., Nakashima, K., Shibata, M., Demura, M. & Kandori, H. Protein-bound water as the determinant of asymmetric functional conversion between light-driven proton and chloride pumps. *Biochemistry* **51**, 4677-4684, (2012).

49 Balashov, S. P. *et al.* Effect of the arginine-82 to alanine mutation in bacteriorhodopsin on dark adaptation, proton release, and the photochemical cycle. *Biochemistry* **32**, 10331-10343, (1993).

50 Balashov, S. P. *et al.* The two pKa's of aspartate-85 and control of thermal isomerization and proton release in the arginine-82 to lysine mutant of bacteriorhodopsin. *Biochemistry* **34**, 8820-8834, (1995).

51 Brown, L. S., Bonet, L., Needleman, R. & Lanyi, J. K. Estimated acid dissociation constants of the Schiff base, Asp-85, and Arg-82 during the bacteriorhodopsin photocycle. *Biophys. J.* **65**, 124-130, (1993).

52 Martinez, L. C. & Turner, G. J. High-throughput screening of bacteriorhodopsin mutants in whole cell pastes. *Biochim. Biophys. Acta* **1564**, 91-98, (2002).

53 Lanyi, J. K., Tittor, J., Váró, G., Krippahl, G. & Oesterhelt, D. Influence of the size and protonation state of acidic residue 85 on the absorption spectrum and photoreaction of the bacteriorhodopsin chromophore. *Biochim. Biophys. Acta* **1099**, 102-110, (1992).

54 Millerd, J. E. *et al.* Improved sensitivity in blue-membrane bacteriorhodopsin films. *Opt. Lett.* **24**, 1355-1357, (1999).

55 Brown, L. S., Needleman, R. & Lanyi, J. K. Interaction of proton and chloride transfer pathways in recombinant bacteriorhodopsin with chloride transport activity: implications for the chloride translocation mechanism. *Biochemistry* **35**, 16048-16054, (1996).

56 Váró, G. *et al.* A residue substitution near the beta-ionone ring of the retinal affects the M substates of bacteriorhodopsin. *Biophys. J.* **61**, 820-826, (1992).

57 Gat, Y., Friedman, N., Sheves, M. & Ottolenghi, M. Interaction between Asp-85 and the proton-releasing group in bacteriorhodopsin. A study of an O-like photocycle intermediate. *Biochemistry* **36**, 4135-4148, (1997).

58 Needleman, R. *et al.* Properties of Asp212----Asn bacteriorhodopsin suggest that Asp212 and Asp85 both participate in a counterion and proton acceptor complex near the Schiff base. *J Biol Chem* **266**, 11478-11484, (1991).

59 Richter, H. T., Needleman, R. & Lanyi, J. K. Perturbed interaction between residues 85 and 204 in Tyr-185→Phe and Asp-85→Glu bacteriorhodopsins. *Biophys J* **71**, 3392-3398, (1996).

60 Perálvarez-Marín, A. *et al.* Influence of proline on the thermostability of the active site and membrane arrangement of transmembrane proteins. *Biophys. J.* **95**, 4384-4395, (2008).

61 Wang, Y. *et al.* Effect of substitution of proline-77 to aspartate on the light-driven proton release of bacteriorhodopsin. *Photochem. Photobiol.* **88**, 922-927, (2012).

62 Imasheva, E. S. *et al.* Exploring the function of Tyr83 in bacteriorhodopsin: features of the Y83F and Y83N mutants. *Biochemistry* **40**, 13320-13330, (2001).

63 Govindjee, R. *et al.* Effects of substitution of tyrosine 57 with asparagine and phenylalanine on the properties of bacteriorhodopsin. *Biochemistry* **34**, 4828-4838, (1995).

64 Misra, S. *et al.* Mutation of arginine 134 to lysine alters the pK_a_s of key groups involved in proton pumping by bacteriorhodopsin. *Photochem Photobiol* **66**, 774-783, (1997).

65 Perálvarez-Marín, A., Marquez, M., Bourdelande, J. L., Querol, E. & Padrós, E. Thr-90 plays a vital role in the structure and function of bacteriorhodopsin. *J Biol Chem* **279**, 16403-16409, (2004).

66 Brown, L. S., Dioumaev, A. K., Needleman, R. & Lanyi, J. K. Connectivity of the retinal Schiff base to Asp85 and Asp96 during the bacteriorhodopsin photocycle: the local-access model. *Biophys. J.* **75**, 1455-1465, (1998).

67 Brown, L. S., Zimanyi, L., Needleman, R., Ottolenghi, M. & Lanyi, J. K. Photoreaction of the N intermediate of bacteriorhodopsin, and its relationship to the decay kinetics of the M intermediate. *Biochemistry* **32**, 7679-7685, (1993).

68 Soppa, J. *et al.* Bacteriorhodopsin mutants of *Halobacterium sp.* GRB. II. Characterization of mutants. *J. Biol. Chem.* **264**, 13049-13056, (1989).

69 Cao, Y., Brown, L. S., Needleman, R. & Lanyi, J. K. Relationship of proton uptake on the cytoplasmic surface and reisomerization of the retinal in the bacteriorhodopsin photocycle: an attempt to understand the complex kinetics of the pH changes and the N and O intermediates. *Biochemistry* **32**, 10239-10248, (1993).

70 Delaney, J. K., Schweiger, U. & Subramaniam, S. Molecular mechanism of protein-retinal coupling in bacteriorhodopsin. *Proc. Natl. Acad. Sci. USA* **92**, 11120-11124, (1995).

71 Martinez, L. C., Thurmond, R. L., Jones, P. G. & Turner, G. J. Subdomains in the F and G helices of bacteriorhodopsin regulate the conformational transitions of the reprotonation mechanism. *Proteins* **48**, 269-282, (2002).

72 Shimono, K., Iwamoto, M., Sumi, M. & Kamo, N. Effects of three characteristic amino acid residues of *pharaonis* phoborhodopsin on the absorption maximum. *Photochem. Photobiol.* **72**, 141-145, (2000).

73 Iwamoto, M. *et al.* Role of Asp193 in chromophore-protein interaction of pharaonis phoborhodopsin (sensory rhodopsin II). *Biophys. J.* **83**, 1130-1135, (2002).

74 Shimono, K., Ikeura, Y., Sudo, Y., Iwamoto, M. & Kamo, N. Environment around the chromophore in *pharaonis* phoborhodopsin: mutation analysis of the retinal binding site. *Biochim. Biophys. Acta* **1515**, 92-100, (2001).

75 Klare, J. P. *et al.* Probing the proton channel and the retinal binding site of Natronobacterium pharaonis sensory rhodopsin II. *Biophys. J.* **82**, 2156-2164, (2002).

76 Shimono, K. *et al.* Importance of the broad regional interaction for spectral tuning in Natronobacterium pharaonis phoborhodopsin (sensory rhodopsin II). *J. Biol. Chem.* **278**, 23882-23889, (2003).

77 Fan, Y., Solomon, P., Oliver, R. P. & Brown, L. S. Photochemical characterization of a novel fungal rhodopsin from *Phaeosphaeria* nodorum. *Biochim Biophys Acta* **1807**, 1457-1466, (2011).

78 Waschuk, S. A., Bezerra, A. G., Jr., Shi, L. & Brown, L. S. Leptosphaeria rhodopsin: bacteriorhodopsin-like proton pump from a eukaryote. *Proc. Natl. Acad. Sci. USA* **102**, 6879-6883, (2005).

79 Bieszke, J. A., Spudich, E. N., Scott, K. L., Borkovich, K. A. & Spudich, J. L. A eukaryotic protein, NOP-1, binds retinal to form an archaeal rhodopsin-like photochemically reactive pigment. *Biochemistry* **38**, 14138-14145, (1999).

80 Fu, H. Y. *et al.* A novel six-rhodopsin system in a single archaeon. *J. Bacteriol.* **192**, 5866-5873, (2010).

81 Nakao, Y. *et al.* Photochemistry of a putative new class of sensory rhodopsin (SRIII) coded by xop2 of Haloarcular marismortui. *J Photochem Photobiol B* **102**, 45-54, (2011).

82 Sineshchekov, O. A., Li, H., Govorunova, E. G. & Spudich, J. L. Photochemical reaction cycle transitions during anion channelrhodopsin gating. *Proc. Natl. Acad. Sci. USA* **113**, E1993-2000, (2016).

83 Wietek, J., Broser, M., Krause, B. S. & Hegemann, P. Identification of a natural green light absorbing chloride conducting channelrhodopsin from *Proteomonas sulcata*. *J. Biol. Chem.* **291**, 4121-4127, (2016).

84 Balashov, S. P. *et al.* Aspartate-histidine interaction in the retinal schiff base counterion of the light-driven proton pump of *Exiguobacterium sibiricum*. *Biochemistry* **51**, 5748-5762, (2012).

85 Engqvist, M. K. *et al.* Directed evolution of *Gloeobacter violaceus* rhodopsin spectral properties. *J. Mol. Biol.* **427**, 205-220, (2015).

86 Sasaki, K. *et al.* Chimeric proton-pumping rhodopsins containing the cytoplasmic loop of bovine rhodopsin. *PLoS ONE* **9**, e91323, (2014).

87 Sudo, Y. & Yoshizawa, S. Functional and photochemical characterization of a light-driven proton pump from the gammaproteobacterium *Pantoea vagans*. *Photochem. Photobiol.* **92**, 420-427, (2016).

88 Harris, A. *et al.* A new group of eubacterial light-driven retinal-binding proton pumps with an unusual cytoplasmic proton donor. *Biochim. Biophys. Acta* **1847**, 1518-1529, (2015).

89 Ito, S. *et al.* Water-containing hydrogen-bonding network in the active center of channelrhodopsin. *J. Am. Chem. Soc.* **136**, 3475-3482, (2014).

90 Lin, J. Y., Lin, M. Z., Steinbach, P. & Tsien, R. Y. Characterization of engineered channelrhodopsin variants with improved properties and kinetics. *Biophys. J.* **96**, 1803-1814, (2009).

91 Kato, H. E. *et al.* Atomistic design of microbial opsin-based blue-shifted optogenetics tools. *Nat. Commun.* **6**, 7177, (2015).

92 Doi, S. *et al.* Structural and functional roles of the N- and C-terminal extended modules in channelrhodopsin-1. *Photochem. Photobiol. Sci.* **14**, 1628-1636, (2015).

93 Yizhar, O. *et al.* Neocortical excitation/inhibition balance in information processing and social dysfunction. *Nature* **477**, 171-178, (2011).

94 Schneider, F., Grimm, C. & Hegemann, P. Biophysics of channelrhodopsin. *Annu. Rev. Biophys.* **44**, 167-186, (2015).

95 Yizhar, O., Fenno, L. E., Davidson, T. J., Mogri, M. & Deisseroth, K. Optogenetics in neural systems. *Neuron* **71**, 9-34, (2011).

96 Scholz, F., Bamberg, E., Bamann, C. & Wachtveitl, J. Tuning the primary reaction of channelrhodopsin-2 by imidazole, pH, and site-specific mutations. *Biophys. J.* **102**, 2649-2657, (2012).

97 Ritter, E., Stehfest, K., Berndt, A., Hegemann, P. & Bartl, F. J. Monitoring light-induced structural changes of Channelrhodopsin-2 by UV-visible and Fourier transform infrared spectroscopy. *J. Biol. Chem.* **283**, 35033-35041, (2008).

98 Klapoetke, N. C. *et al.* Independent optical excitation of distinct neural populations. *Nat. Methods* **11**, 338-346, (2014).

99 Zhang, F. *et al.* The microbial opsin family of optogenetic tools. *Cell* **147**, 1446-1457, (2011).

100 Smolensky Koganov, E., Hirshfeld, A. & Sheves, M. Retinal beta-ionone ring-salinixanthin interactions in xanthorhodopsin: a study using artificial pigments. *Biochemistry* **52**, 1290-1301, (2013).

101 Kamo, N. *et al.* A light-driven proton pump from *Haloterrigena turkmenica*: functional expression in *Escherichia coli* membrane and coupling with a H^+^ co-transporter. *Biochem. Biophys. Res. Commun.* **341**, 285-290, (2006).

102 Kim, S. Y., Waschuk, S. A., Brown, L. S. & Jung, K. H. Screening and characterization of proteorhodopsin color-tuning mutations in *Escherichia coli* with endogenous retinal synthesis. *Biochim. Biophys. Acta* **1777**, 504-513, (2008).
